# High Creatine Kinase (CK)-MB and Lactate Dehydrogenase in the Absence of Myocardial Injury or Infarction: A Case Report

**DOI:** 10.5681/jcvtr.2014.014

**Published:** 2014-03-21

**Authors:** Nasrollah Maghamiour, Naser Safaie

**Affiliations:** ^1^Department of Cardiac Surgery, Behsat Hospital, School of Medical Science, Tehran, Iran; ^2^Cardiovascular Research Center, Tabriz University of Medical Sciences, Tabriz, Iran

**Keywords:** Creatine Kinase, Macro Creatine Kinase, Myocardial Infarction

## Abstract

Acute myocardial infarction (AMI) is a life threatening condition that needs emergency diagnosis and early treatment in the emergency room. Rapid laboratory testing for creatine kinase (CK)-MB greatly revolutionized the diagnosis and management of acute myocardial infarction. We report a case with chest pain that referred to the emergency department (ED). Laboratory data showed high serum levels of creatine kinase and lactate dehydrogenase. With diagnosis of acute myocardial infarction, he was hospitalized and angiography was performed which showed three vessels disease; the patient was referred to surgical ward for coronary artery bypass graft. Surgery was performed after one week; during the operation there was no sign of infarction over the heart. Our observation suggests that false positive laboratory result may be due to other condition which must be evaluated.

## Introduction

With rapid laboratory testing for creatine kinase (CK)-MB and lactate dehydrogenase, early diagnosis and management of acute myocardial infarction (AMI) is performed in the emergency room. However, there are conditions that are known to lower the sensitivity and specificity of CK-MB activity assays. False-positive results may be seen in renal failure patients, concurrent skeletal muscle and myocardial injuries, and many other conditions, such as non -cardiac surgery, chest trauma, asthma, malignancies and pulmonary embolism.^1^ High CK-MB activity in the absence of myocardial injury is due to presence of macroenzyme )macro) CK in the serum of these patients.

## Case Presentation

A 64 year old man presented to our ED because of chest pain for an hour. Physical examination revealed no significant finding. However, EKG showed T inversions in pericardial leads. Laboratory investigations were within normal levels except for very high CKMB and CPK and lactate dehydrogenase. Chest x ray was normal. Echocardiography was normal with ejection fraction of 60%. Patient was treated for acute myocardial infarction and hospitalized for angiography. Angiography revealed three vessel diseases, and consulted for coronary artery bypass surgery. In surgical ward patient chest pain improved but CK-MB and CPK and lactate dehydrogenase were very high. After one week of hospitalization patient underwent coronary artery bypass operation. During the operation there was no sign of myocardial infarction over the myocardium. Patient tolerated the operation without any event. After the operation CK-MB and CPK titer did not decrease, and patient was discharged from the hospital.

## Discussion

Elevated CK and CK-MB activity often leads to suspicion of AMI or ACS, especially when patients present with chest symptoms mimicking angina pectoris and the presence of macro CK can be troublesome. Although currently, the diagnostic confusion of AMI/ACS with macro CK can be resolved by careful identification of macro CK or by the determination of troponin levels.^2^ In areas where macro CK measurement or troponin assays are not available, CK and CK-MB activity assays are still reliable, and the identification of macro CK avoids unnecessary and costly diagnostic procedures. Macro CK can usually be recognized on CK electrophoresis. So far, only two kinds of macro-creatine have been discovered. BB isoenzyme of the enzyme attached to immunoglobulin which is mainly immunoglobulin G is seen in type one whose manifestation is related to autoimmune causes. Being a polymer of mitochondrial creatine kinase, the second type can be detected in tumors.^3^ Often, macro CK causes only a mild elevation in CK or a high CK-MB/CK ratio with a normal level of total CK. Clinically, the absence of symptoms, the presence of symptoms atypical for the abnormal level of CK, or an isolated and persistently increased CK favor the presence of macro CK. In our case, the measured CK-MB activity was very high about 800 IU/L and higher than total CPK. Our patient had ulcerative colitis who was on medical treatment for several years. Rarely, under certain pathological conditions, abnormally increased CK-BB may contribute to the falsely elevated CK-MB activity that may exceed total CK activity. High level of CK-MB is seen in patient with a history of asymptomatic chronic hepatitis C without cryoglobulinemia, but mildly elevated liver enzymes.^4^ In the follow-up of these patients the cardiac biochemistry profile showed persistent mildly elevated CK-MB. An increase in total creatine kinase activity with the creatine kinase MB fraction value exceeding the total creatine kinase activity is seen in thrombo-embolic disease. Values for creatine kinase MB fraction in the immunoinhibition assay may be due to the existence of macro creatine kinase type I in ulcerative colitis.^5^ Elevated CK-MB fraction is seen in prostatic carcinoma and other underlying malignancy, such as breast cancer. Certain drugs that can increase CPK measurements include amphotericin B, certain anesthetics, statins, fibrates, dexamethasone, alcohol, and cocaine.

Macro CK 1 has been reported to be associated with a variety of diseases, including hypothyroidism, malignancies, autoimmune diseases, myositis, and cardiovascular disease. More rarely, macro CK 1 has been described in patients with irritable bowel syndrome and bronchopulmonary chronic illness. Macro CK type 2 is frequently found in patients who are critically ill or who have widespread tissue damage such as severe liver disease and disseminated malignancies.^6^ In general, the presence of macro CK type 2 may be viewed as a warning signal of occult malignancies, a poor prognostic sign in patients with a malignancy, or a reflection of the severity of an underlying illness.^7^ In conclusion, macro CK can be detected as an incidental finding in healthy individuals or as a marker of certain diseases (autoimmune diseases, cancer, severe liver disease, and serious illness). It is particularly important to recognize macro CK in patients with symptoms mimicking ACS to avoid unnecessary specialist consultations and invasive procedures. Despite the usefulness of troponin assays, confirmation is required to completely replace CK and CK isoenzymes by troponins in AMI/ACS diagnosis. It is important for clinicians to understand the biochemistry and clinical significance of macro CK in the context of modern laboratory medicine. Injury or stress to muscle tissue, the heart, or the brain can be associated with increased total CPK levels^8^ due to CPK leakage into the circulation. Increase in any particular type of CPK would define the type of damaged tissue. Some factors including cardiac catheterization, intramuscular injections, trauma to muscles, recent surgery, and heavy exercise might influence test results.

## Acknowledgments

We thank our colleagues in the Section of Biochemistry of the Department of Laboratory Medicine for excellent consultation and assistance with biochemical testing.

## Ethical issues

The study was approved by our local Ethics Committee.

## Competing interests

The author declares that he has no competing interests.
